# Supplementary material for: Modified Graphene Oxide-Incorporated Thin-Film Composite Hollow Fiber Membranes through Interface Polymerization on Hydrophilic Substrate for CO2 Separation
Source: Membranes (Basel). 2021 Aug 25;11(9):650. doi: 10.3390/membranes11090650 (PMC8470957; doi:10.3390/membranes11090650)
Supplement: Supplementary file 1 [file membranes-11-00650-s001.zip › membranes-1343531-supplementary.pdf]

membranes

Supplementary Material

Modified Graphene Oxide-Incorporated Thin-Film Composite Hollow Fiber Membranes through Interface Polymerization on Hydrophilic Substrate for CO_2 Separation

Ook Choi [1,†], Iqubal Hossain [1,2,†], Insu Jeong [1,2], Chul-Ho Park [3], Yeonho Kim [1] and Tae-Hyun Kim [1,2,*]

1 Research Institute of Basic Sciences, Incheon National University, 119 Academy-ro, Yeonsu-gu, Incheon 22012, Korea; ooksclub@inu.ac.kr (O.C.); iqubal.chem.ru.08@gmail.com (I.H.); jis_3088@naver.com (I.J.); yeonho@inu.ac.kr (Y.K.)
2 Organic Material Synthesis Laboratory, Department of Chemistry, Incheon National University, 119 Academy-ro, Yeonsu-gu, Incheon 22012, Korea
3 Jeju Global Research Center (JGRC), Korea Institute of Energy Research (KIER), 200 Haemajihaean-ro, Gujwa-eup 63357, Jeju Specific Self-Governing Province, Korea; chpark@kier.re.kr
* Correspondence: tkim@inu.ac.kr; Tel.: +82-32-8358232
† These authors contributed equally to this work.

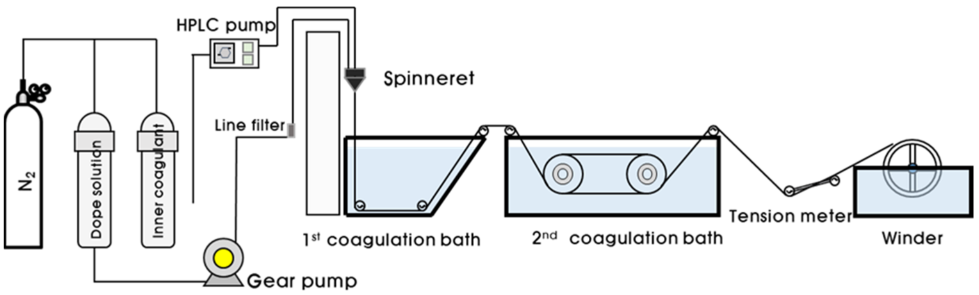

Figure S1. Schematic diagram of hollow fiber spinning process.

Permeate gas flux measurement

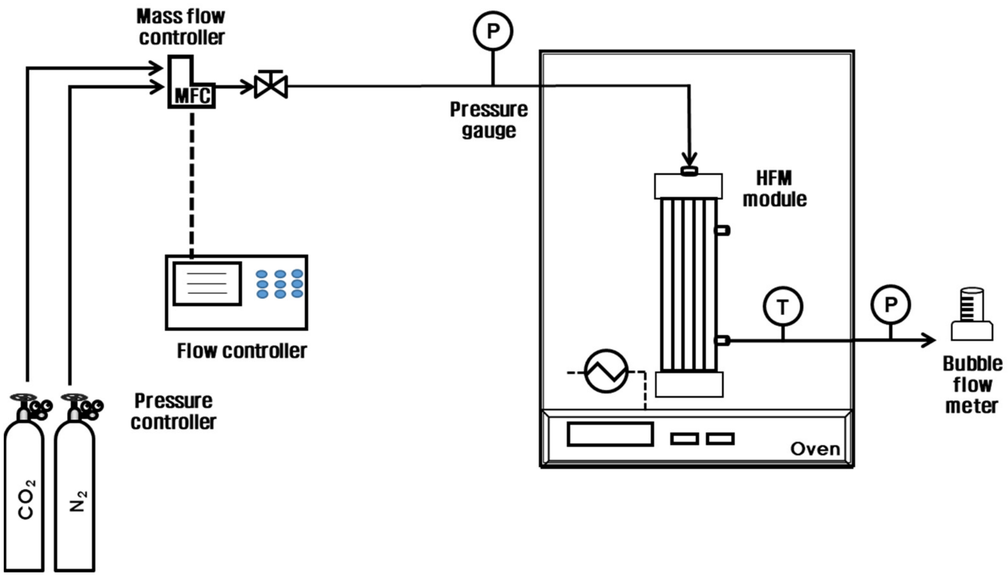

Figure S2. Schematic diagram of gas permeation experiment apparatus.

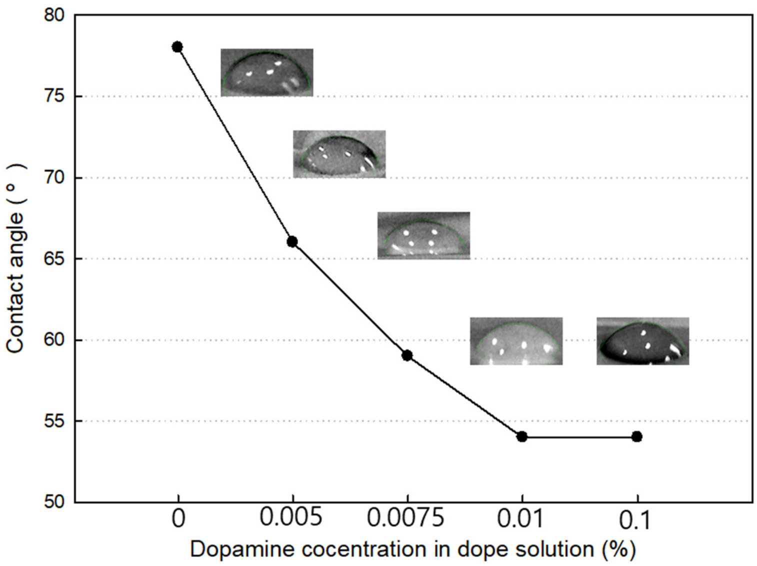

Figure S1. Effect of dopamine concentrations on contact angle changes.

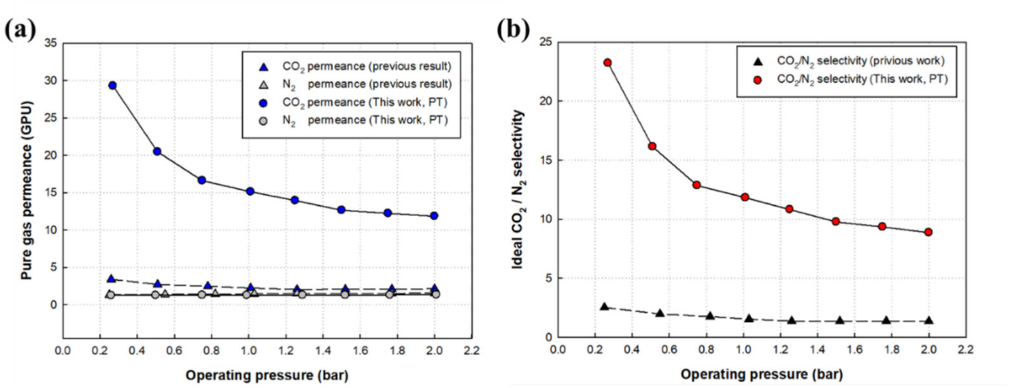

Figure S4. Comparison between previous work results and this work against gas permeance and ideal gas selectivity, showing the effect of SDS on the TFN composite membrane.

Hollow fiber membrane surface interfacial energy

The surface characteristics of hollow fiber membranes were examined by measuring the surface interfacial energy. The interfacial energy was determined based on the AFM results and contact angle test results on the membrane surface using the Young-Dupre equation below, and the results are presented in Table S1.

$$-\Delta G_{SL} = \gamma_L [1 + \cos\theta/\Delta]$$

Here, γL is the liquid surface tension (D.I water: γ_L = 72.4 mJ/m^2), θ is the contact angle, and Δ is the relative surface area (Δ = the actual surface area divided by the planar area (3 × 3, from AFM analysis) [S1].

Table S1. Surface roughness and contact angle measurement of TFC hollow fiber membranes.

**Membrane**	**R_q (nm)**	**R_a (nm)**	**Contact angle (°)**	**Relative surface area (Δ)**	**$-\Delta G_{SL}$ (mJ/m^2)**
PT	1.77	1.44	44.2±1.5	1.005	124.0
PTS 0.1	1.98	1.53	42.5±0.5	1.006	125.5
PTS 0.3	3.81	2.96	37.3±1.2	1.010	129.4
PTS 0.5	4.36	3.52	33.1±1.1	1.011	132.4

R_a = average surface roughness, R_q = root mean square surface roughness, $-\Delta GSL$ = solid-liquid interfacial free energy.

Table S2. Summary of CO2 permeance and CO2/N2 selectivity MMMs membranes results.

**Type**	**Polymer (membrane type, operating pressure)**	**Permeance Of CO_2 (GPU)**	**CO_2/N_2 Selectivity (-)**	**Ref.**

NH_2-SiO_2 (Silica)	PEBAX 1657 (flat sheet, 5 bar)	119	25.85	[S2]
ETS-4 (Tit.silicate)	PES (HFM, 0.25 bar)	17	33	[S3]
PDA-ZIP-8 (Zeolite)	PVAm (flat sheet)	297	83	[S4]
APTMS-MIL-53 (MOF)	Ultem (flat sheet, 5 bar)	17.8	41.8	[S5]
Cu-MOF2 (MOF)	Polyozazoline (flat sheet)	3	55	[S6]
UIO-66-NH_2 (MOF)	Pebax-1657 (flat sheet)	338	57	[S7]
MIL-53+NHCOH (MOF)	Pebax-1657 (flat sheet, 2 bar)	4.6	65.02	[S8]
CuBDC (MOF)	Polyactive (flat sheet , 2 bar)	3.3	66	[S9]
Amin-GO (GO)	PES (flat sheet, 2 bar)	13.26	37.88	[S10]
GO (GO)	PSf (5bar)	65.24	17.26	[S11]
PGO (GO)	PES-PEG-Dopa	73	60	This work
β-CD-MWCNT (CNT)	CA (flat sheet, 3 bar)	400.93	32.92	[S12]
f-MWCNT (CNT)	PES (HFM, 0.25 bar)	21	27.35	[S13]

References

1. [S1] R.N. Wenzel, Surface Roughness and Contact Angle, J. Phys. Colloid Chem., 53 (1949), pp.1466–1467, 10.1021/j150474a015
2. [S2] M. Wang, Z. Wang, N. Li, J. Liao, S. Zhao, J. Wang, S. Wang, Relationship between polymer-filler interfaces in separation layers and gas transport properties of mixed matrix composite membranes, J. Memb. Sci., 495 (2015), pp.252-268, 10.1016/j.memsci.2015.08.019
3. [S3] O. Choi, Y. Kim, J.D. Jeon, T.H. Kim, Preparation of thin film nanocomposite hollow fiber membranes with polydopamine-encapsulated Engelhard titanosilicate-4 for gas separation applications, J. Memb. Sci., 650 (2021), pp.118946-118956, 10.1016/j.memsci.2020.118946
4. [S4] S. Zhao, X. Cao, Z. Ma, Z. Wang, Z. Qiao, J. Wang, S. Wang, Mixed-Matrix Membranes for CO2/N2 Separation Comprising a Poly(vinylamine) Matrix and Metal-Organic Frameworks, Ind. Eng. Chem. Res., 54 (2015), pp.5139-5148, 10.1021/ie504786x
5. [S5] H. Zhu, L. Wang, X. Jie, D. Liu, Y. Cao, Improved Interfacial Affinity and CO2 Separation Performance of Asymmetric Mixed Matrix Membranes by Incorporating Postmodified MIL-53(Al), ACS Appl. Mater. Interfaces., 8 (2016), pp.22696-22704, 10.1021/acsami.6b07686
6. [S6] S.Y. Lim, J. Choi, H.Y. Kim, Y. Kim, S.J. Kim, Y.S. Kang, J. Won, New CO2 separation membranes containing gas-selective Cu-MOFs, J. Memb. Sci., 467 (2014), pp.67-72, 10.1016/j.memsci.2014.05.014
7. [S7] Y. Jiang, C. Liu, J. Caro, A. Huang, A new UiO-66-NH2 based mixed-matrix membranes with high CO2/CH4 separation performance, Microporous Mesoporous Mater., 274 (2019), pp.203-211, 10.1016/j.micromeso.2018.08.003
8. [S8] Q. Li, J. Duan, W. Jin, Efficient CO2/N2 separation by mixed matrix membrane with amide functionalized porous coordination polymer filler, Chinese Chem. Lett., 29 (2018), pp.854-856, 10.1016/j.cclet.2017.11.008
9. [S9] A. Sabetghadam, X. Liu, S. Gottmer, L. Chu, J. Gascon, F. Kapteijn, Thin mixed matrix and dual layer membranes containing metal-organic framework nanosheets and Polyactive™ for CO2 capture, J. Memb. Sci., 570-517 (2019), pp.226-235., 10.1016/j.memsci.2018.10.047
10. [S10] S. Ebrahimi, S. Mollaiy-Berneti, H. Asadi, M. Peydayesh, F. Akhlaghian, T. Mohammadi, PVA/PES-amine-functional graphene oxide mixed matrix membranes for CO2/CH4 separation: Experimental and modeling, Chem. Eng. Res. Des., 109 (2016), pp.647-656, 10.1016/j.cherd.2016.03.009
11. [S11] K. Zahri, K.C. Wong, P.S. Goh, A.F. Ismail, Graphene oxide/polysulfone hollow fiber mixed matrix membranes for gas separation, RSC Adv., 6 (2016), pp.89130-89139, 10.1039/c6ra16820e
12. [S12] A.L. Ahmad, Z.A. Jawad, S.C. Low, S.H.S. Zein, A cellulose acetate/multi-walled carbon nanotube mixed matrix membrane for CO2/N2 separation, J. Memb. Sci., 451 (2014), 55-66, 10.1016/j.memsci.2013.09.043
13. [S13] O. Choi, S. Karki, R.R. Pawar, S. Hazarika, P.G. Ingole, A new perspective of functionalized MWCNT incorporated thin film nanocomposite hollow fiber membranes for the separation of various gases, J. Environ. Chem. Eng., 9 (2021), pp.104774-104784, 10.1016/j.jece.2020.104774
